# Analysis of Beta-Cell Gene Expression Reveals Inflammatory Signaling and Evidence of Dedifferentiation following Human Islet Isolation and Culture

**DOI:** 10.1371/journal.pone.0030415

**Published:** 2012-01-27

**Authors:** Sarita Negi, Arif Jetha, Reid Aikin, Craig Hasilo, Rob Sladek, Steven Paraskevas

**Affiliations:** 1 Human Islet Transplantation Laboratory, McGill University Health Centre, Montreal, Quebec, Canada; 2 Department of Surgery, McGill University, Montreal, Quebec, Canada; 3 McGill University and Genome Quebec Innovation Centre, Montreal, Quebec, Canada; University of British Columbia, Canada

## Abstract

The stresses encountered during islet isolation and culture may have deleterious effects on beta-cell physiology. However, the biological response of human islet cells to isolation remains poorly characterized. A better understanding of the network of signaling pathways induced by islet isolation and culturing may lead to strategies aimed at improving islet graft survival and function. Laser capture microdissection (LCM) was used to extract beta-cell RNA from 1) intact pancreatic islets, 2) freshly isolated islets, 3) islets cultured for 3 days, and changes in gene expression were examined by microarray analysis. We identified a strong inflammatory response induced by islet isolation that continues during *in-vitro* culture manifested by upregulation of several cytokines and cytokine-receptors. The most highly upregulated gene, interleukin-8 (IL-8), was induced by 3.6-fold following islet isolation and 56-fold after 3 days in culture. Immunofluorescence studies showed that the majority of IL-8 was produced by beta-cells themselves. We also observed that several pancreas-specific transcription factors were down-regulated in cultured islets. Concordantly, several pancreatic progenitor cell-specific transcription factors like SOX4, SOX9, and ID2 were upregulated in cultured islets, suggesting progressive transformation of mature beta-cell phenotype toward an immature endocrine cell phenotype. Our findings suggest islet isolation and culture induces an inflammatory response and loss of the mature endocrine cell phenotype. A better understanding of the signals required to maintain a mature beta-cell phenotype may help improve the efficacy of islet transplantation.

## Introduction

Islet transplantation is a potential treatment for type1 diabetes but is limited by insufficient transplantable beta-cell mass and functional impairment after transplantation [1]. Even with available donor organs, the current method of islet isolation and culture results in islet cell loss by cell death and dedifferentiation [2], [3]. Furthermore, the injury response of islet cells due to isolation may have negative consequences at the graft site. Our objective is to characterize the response of human beta-cells to isolation and culture in order to better maintain islets in culture and at the graft site.

Islet isolation exposes these cells to a number of stresses that can adversely affect cell survival [4]. While various strategies have been explored over the past decade to improve isolated islet cell survival, there has yet to be an approach to prevent islet cell death that has translated successfully into clinical use. A better understanding of the network of signaling pathways induced by islet isolation and culture may lead to better strategies aimed at preventing islet cell death.

Current protocols in islet isolation and transplantation can result in grafts with elevated immunogenic properties [5] which may adversely affect primary graft function. The recipient's innate inflammatory response to islet grafts, known as, the immediate blood-mediated inflammatory response (IBMIR), has been suggested to cause loss of the transplanted islet cells [6], [7]. Upon injection into the recipient, direct exposure of human islets to blood triggers IBMIR, characterized by platelet aggregation, complement activation and infiltration of islets with neutrophils and monocytes. Furthermore, it has been shown that islets promote inflammation through their release of chemoattractants like Tissue Factor (TF) and MCP1 [8]. The influence of human islet isolation stress on expression of TF, MCP1 or other proinflammatory mediators, is not well studied.

Evidence suggests that the beta-cell phenotype is fragile and easily lost upon removal of these cells from their native environment [3], [9]. It has been demonstrated that beta-cells from dispersed isolated human islets undergo an epithelial-to-mesenchymal transition in culture [10], and it is thought that the same process occurs in beta-cells from whole islets [11]. How human islet isolation and culturing alters the signals controlling pancreatic endocrine cell fate is not known.

**Figure 1:**
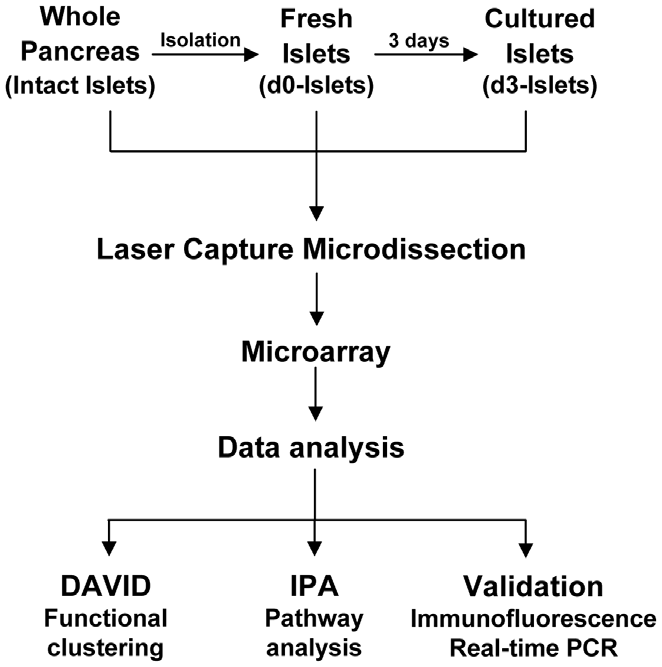
Schematic diagram of the workflow. Frozen sections of the intact human pancreas, freshly isolated islets (d0) and cultured islets (d3) were processed for LCM. The beta-cells were identified by their intrinsic autofluorescence and captured by LCM, followed by RNA extraction, amplification and labeling. Labeled RNA was hybridized to Human WG-6 Expression Arrays (Illumina) and scanned by BeadArray Reader. Expression data were analyzed by Flexarray, DAVID and IPA and validated by immunostaining.

Previous studies on isolated human islets were often limited by the inability to compare findings in cultured islets to those within the intact pancreas. Recently, laser capture microdissection (LCM) was shown to be a viable method to obtain beta-cell enriched samples for gene expression analysis from whole pancreas sections [12]. In this study, our goal was to assess the genome-wide effect of islet isolation and culture on beta-cell gene expression, using beta-cells from intact pancreas as the reference point (Figure 1). LCM was used to collect beta-cells from 1) intact pancreas, 2) freshly isolated and 3) cultured islets, which allowed us to observe the response of these cells to isolation and culture.

## Materials and Methods

### Pancreas recovery, islet isolation and *in-vitro* culture

Human pancreata (n = 8) were recovered from multi-organ deceased donors after appropriate consent was obtained by personnel from Quebec-Transplant. Before isolation, a wedge biopsy was embedded in Tissue-TEK OCT (Sakura Finetek), snap frozen in liquid nitrogen and stored at −80°C. Islet isolation was performed at the Human Islet Isolation Laboratory, McGill University Health Centre, according to the protocol previously described by Ricordi [13]. Immediately following isolation, 2500 islet equivalents (IE) were embedded in Tissue-TEK OCT, snap frozen in liquid nitrogen and stored at −80°C. We also placed 5000 IE in culture in CMRL-1066 (Invitrogen) supplemented with 10% FBS, Penicillin/Streptomycin (100 µg/mL)(Wisent), and Fungizone (Invitrogen) at 37°C. Culture medium was changed every other day. On the third day, the cultured islets were embedded in Tissue-TEK OCT, snap frozen in liquid nitrogen and stored at −80°C. All the procedures using isolating human islets were approved by the McGill University Health Centre Research Ethics Board. The average yield and purity of islets after isolations was 227,379±15,224 IE and 87.8±2.4%, respectively. Islet viability was assessed using SYTO-13 staining and was 96.5±1.2%, following isolation, viability was stable through the 3 days in culture.

### Laser capture microdissection

Samples were divided into three groups: 1) beta-cells from intact islets in whole pancreas tissue (n = 8), 2) beta-cells from freshly isolated islets (n = 8, d0), and 3) beta-cells from islets cultured for three days (n = 5, d3). Ten micron frozen sections for each of these samples were prepared. Immediately before LCM, frozen sections were dehydrated and air dried. LCM was performed using the PixCell II Laser Capture Microdissection System (Arcturus Engineering) by melting thermoplastic films mounted on LCM caps (Arcturus) on beta-cells, identified by their intrinsic autofluorescence as previously described [12] (Figure S1). The cap containing the microdissected cells was then incubated in RLT-plus buffer (Qiagen) with 5% beta-mercaptoethanol. In order to avoid excessive sample rewarming and RNA degradation, each microdissection session was performed in less than 45 minutes, during which a maximum of four sections were processed. For beta-cells from intact pancreas tissue, a minimum of 30 sections were used to obtain sufficient RNA for amplification. For beta-cells extracted from isolated islets, 2000 pulses were needed for sufficient RNA amplification (Figure S2).

### RNA purification and amplification

Total RNA was purified from extracted cells using the Qiagen RNeasy micro-plus kit (Qiagen). RNA quantity and purity were evaluated using a NanoDrop 2000 (NanoDrop Technologies) and Agilent Bioanalyzer, respectively. Samples with RNA integrity number (RIN) values >4 and concentrations >200 pg/µl were deemed acceptable for amplification. RNA was amplified using the Ovation Pico WTA System (Nugen) followed by biotinylation using UNG enzyme (Epicentre Biotech.).

### Microarray Analysis

Labeled RNA (50 ng) was hybridized to Human WG-6 Expression Arrays (Illumina). These chips contain 48,000 probe sets representing 24,000 genes. Array data was normalized by lumi and statistically analyzed by ANOVA using the FlexArray software package (McGill University and Genome Quebec Innovation Centre). Differentially expressed genes were characterized as having a fold-change>2 relative to intact islets and ANOVA p-value<0.05. A hierarchical cluster map was generated for the 359 highly differentially expressed genes using TM4 Microarray Software Suite MultiExperiment Viewer v4.6 (http://www.tm4.org/mev.html). Genes that were differentially expressed were also grouped into functional classes using the Database for Annotation, Visualization and Integrated Discovery (DAVID) 2008 functional annotation tool. In order to determine specific pathways that might be activated in islets, data was analyzed using Ingenuity Pathway Assist [14] (Ingenuity® Systems, www.ingenuity.com). Microarray data has been submitted to the GEO repository (GSE29113).

### Principal components analysis (PCA)

PCA was performed using Flexarray software. This was accomplished by reducing the dimensionality of multivariate data into a multi-dimensional space, allowing for clear visualization of the variation between different samples types.

### Immunofluorescence staining

Frozen sections containing intact pancreas or isolated islets were fixed with acetone for 10 min. After blocking with 5% normal goat serum, sections were incubated with mouse anti-human IL8 (1∶50) (Abcam) and rabbit anti-human insulin or glucagon antibodies (1∶200) (SantaCruz Biotech). The sections were incubated at 4°C overnight. After washing, sections were incubated with anti-mouse-Cy5 and anti-rabbit-FITC. For negative controls, primary antibody was replaced with appropriate serum.

### Identification of NFKB binding sites

Nuclear factor kappa-B (NFKB) binding sites were identified in the promoter region of selected cytokines and top upregulated genes by screening an online database created by Dr. Thomas Gilmore at Boston University (http://people.bu.edu/gilmore/nf-kb/) and http://bioinfo.lifl.fr/NF-KB/.

### Quantitative Real-Time PCR

RNA from laser captured cells from three samples for intact islets, d0-islets and d3-islets were used for real-time PCR. A total of 5 ng of RNA was reverse-transcribed using iScript cDNA synthesis kit (BioRad) according to the manufacturer's instructions. The selected target genes and the primers are listed in Table S2. Real-time PCR was performed using a MyiQ2 real-time detection system (BioRad) and iQ Sybergreen Supermix (BioRad). The expression of housekeeping genes, ribosomal protein S16 (RPS16) or ribosomal protein S18 (RPS18) was also assayed in all samples as an internal reference. Expression values were normalized, using the S16 or S18 RNA value. Fold changes were calculated using 2 ^−ΔΔCt^ (Livak) Method. Reactions in which the reverse transcriptase enzyme was omitted were used as negative controls. The specificity of the PCR amplicons was confirmed using melt curves and by resolving PCR products in a 1.5% agarose gel.

### Statistical analysis

Statistical analysis was performed using single factor ANOVA and p-value less than 0.05 was considered significant. The p-values were determined by unpaired student's t-test. Results are expressed as the means and error bars represent standard deviations from the mean.

## Results

### Microarray analysis of human islet response to isolation and 3 day culture

RNA samples of dissected, beta-cell-enriched tissue from intact islets, freshly isolated islets (d0-islets) and 3 day cultured islets (d3-islets) were analyzed by microarray analysis. Expression analysis detected 88 differentially expressed transcripts in d0-islets compared to intact islets, and 919 differentially expressed transcripts in beta-cells from d3-islets compared to intact islets (>2-fold, p<0.05) (Table 1).

**Table 1:** Summary of microarray analysis result.

Total number of probes: 48000 (24000 human genes)		
Cut-off Criteria	Number of differentially expressed genes	
	d0-Islets	d3-Islets
**Fold change ≥2, p≤0.05**	88	919
Up-regulated genes	57	562
Down-regulated genes	31	357
**Fold change ≥1.5, p≤0.05**	581	2151
Up-regulated genes	428	1386
Down-regulated genes	153	765

Gene-lists were created using genes with p-values≤0.05 and fold changes either ≥2 or ≥1.5 relative to intact islets.

**Figure 2:**
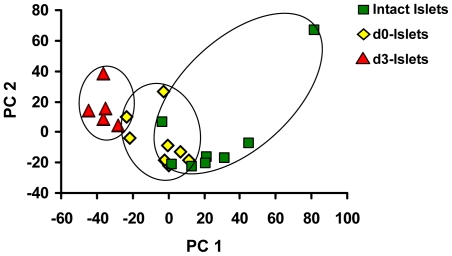
Principal Component analysis (PCA) plot of variability in whole genome expression from intact or isolated islets. Samples were distributed by their similarity in expression data using dimensionality reduction. Pancreatic intact islets (green dots) cluster at right whereas samples from d3-islets (red dots) cluster at left, apart from other groups. The freshly isolated islet samples (yellow dots) cluster at middle and partially intermix with other groups. Principal component 1 and Principal component 2 together accounted for 27% variation.

A PCA plot was generated to determine the distribution of the samples according to their expression pattern (Figure 2). Analysis of the global expression profiles of the samples by PCA revealed a strong correlation of expression data with sample type. We observed distinct separation of the beta-cell enriched samples from intact islets and d3-islets whereas d0-islets appeared as an intermediate cluster, in the 2-dimensional PCA plot. Thus, the variation in expression profile appears to progress with time as suggested by the increasing number of differentially expressed genes with d0- and d3-islets.

**Figure 3:**
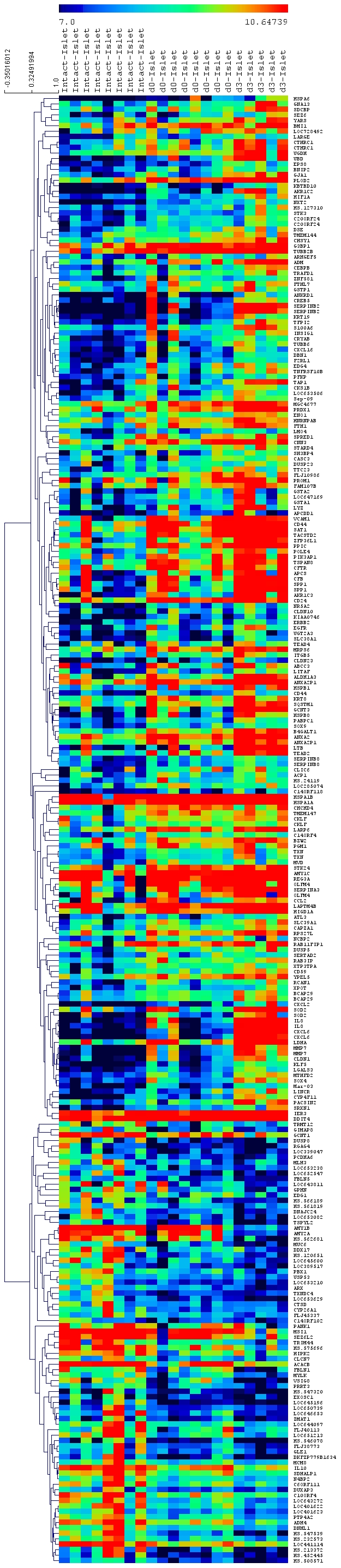
Hierarchical clustering of differentially expressed genes in fresh (d0) and cultured islets (d3). The expression data for intact islets and isolated islets (d0-islets & d3-islets) were analyzed and top 359 genes with p<0.05 and fold change >2.5 were used to create cluster map.

The expression data set for the top 359 differentially expressed genes in d3 vs. intact beta-cells were used to generate a hierarchical cluster map which shows distinct expression patterns for intact-islets, d0-islets and d3-islets (Figure 3). Taken together, these analyses indicate that human islets undergo a measurable change in gene expression in response to isolation and culture.

### Isolation and culturing induces genes involved in inflammation and apoptosis

To understand the biological processes affected due to isolation and culture of islets, we performed Gene Ontology (GO) term enrichment analysis of the gene lists from d0-islets and d3-islets using the DAVID functional annotation tool. Functional clustering revealed that majority of the induced genes in both categories (d0 and d3) were involved in inflammation and apoptosis (Table 2). Cultured islets had a higher number of induced genes for each category and also showed upregulation of genes associated with oxidative stress, cell growth, angiogenesis and IKB/NFKB signaling cascade. Moreover d3-islets also showed upregulation of genes involved in glucose metabolism e.g. TPI1, LDHA, PGM1, HK2, PFKP, PGK1 and ENO1. Interestingly, d3-islets also showed down-regulation of genes required for responses to insulin and glucose stimuli such as LEP, SORBS1, CCND2, IL10, PIK3R1, TXNIP, PFKFB2 and SLC30A8. Furthermore, our data analysis also showed upregulation of 39 cytokines/chemokines and 18 cytokine receptors (Table S1) in d3 islets suggesting activation of cytokine-cytokine receptor pathways.

**Table 2:** Statistically significant altered Biological Processes in islets.

Enriched GO Term	d0-Islets		d3-Islets	
	Fold enrichment^a^	No. of genes	Fold enrichment^a^	No. of genes
**Upregulated Genes**				
Inflammation	24	9	3.9	63
Apoptosis	6.1	9	2.4	55
Regulation of cell proliferation	3.5	9	3.1	27
mRNA catabolic process	21.9	4	3.3	14
Vasculature development/angiogenesis	5.1	4	2.5	18
Regulation of protein kinase cascade	-	-	2.4	26
Regulation of IKB kinase/NFKB cascade	-	-	4	11
Oxidative stress	-	-	3.8	23
Regulation of cell growth/size	10	4	2.6	19
carbohydrate metabolism/Glycolysis	-	-	5.3	27
**Downregulated Genes**				
Transcription	2.5	3	1.1	36
Response to organic substance	-	-	2.1	20
Response to insulin stimulus	-	-	4	5
Response to glucose stimulus	-	-	5.3	3

The differentially expressed genes (p<0.05, fold change>2) from d0-islets and d3-islets were classified into functional groups using DAVID.

GO Term: Gene Ontology Term.

a : Fold enrichment: the overall enrichment score of the biological annotation term based on the associated genes.

The top 10 most highly expressed genes were analyzed in each group using a combination of Flexarray and DAVID. Five of the genes, IL-8, SERPINB2, CFB, OLFM4 and SAT1 were common among the top ten genes from d0 and d3-islets, therefore a total of 15 genes were analyzed for their functions (Table 3). The majority of these genes are involved in the inflammatory response. Other functional classifications include apoptosis, metabolism, cellular stress, cell proliferation and differentiation.

**Table 3:** Function, ANOVA p-values and fold changes of the top ten genes upregulated in freshly isolated (d0) and 3-days cultured islets (d3).

Gene symbol	Function	p-value	Fold Change d0 Islets	Fold Change d3 Islets
IL-8^a^	Inflammation/Angiogenesis	6.1×10^−7^	3.4	56.2
CXCL6	Inflammation	5.8×10^−7^	1.6	30.2
SERPINB2^a^	Inflammation/Apoptosis	1.7×10^−5^	3.1	24.9
CFB^a^	Inflammation	1.3×10^−5^	5.3	16.5
LTB	Inflammation/Apoptosis	4.5×10^−5^	1.7	14.3
GCNT3	Glycosylation	2.5×10^−5^	2.0	12.7
OLFM4^a^	Cell Adhesion	3.6×10^−3^	3.3	12.6
UBD	Proteolysis/Apoptosis	3.4×10^−6^	2.6	11.5
AKR1C3	Fatty acid Metabolism	3.0×10^−5^	2.3	10.9
SAT1^a^	Arginine & proline metabolism	5.9×10^−7^	3.3	10.5
TACSTD2	Signal transduction/cell proliferation	4.9×10^−5^	3.1	7.6
REG3A	Differentiation	3.4×10^−2^	3.3	5.9
VCAM1	Inflammation	3.6×10^−4^	2.9	5.5
HSPA1B	Cellular stress	3.4×10^−3^	3.5	3.2
HSPA1A	Cellular stress	4.2×10^−4^	3.7	2.8

a Genes common between top 10 upregulated genes from d0-islets and d3-islets.

### IL-8 is strongly upregulated in beta-cells in isolated islets

**Figure 4:**
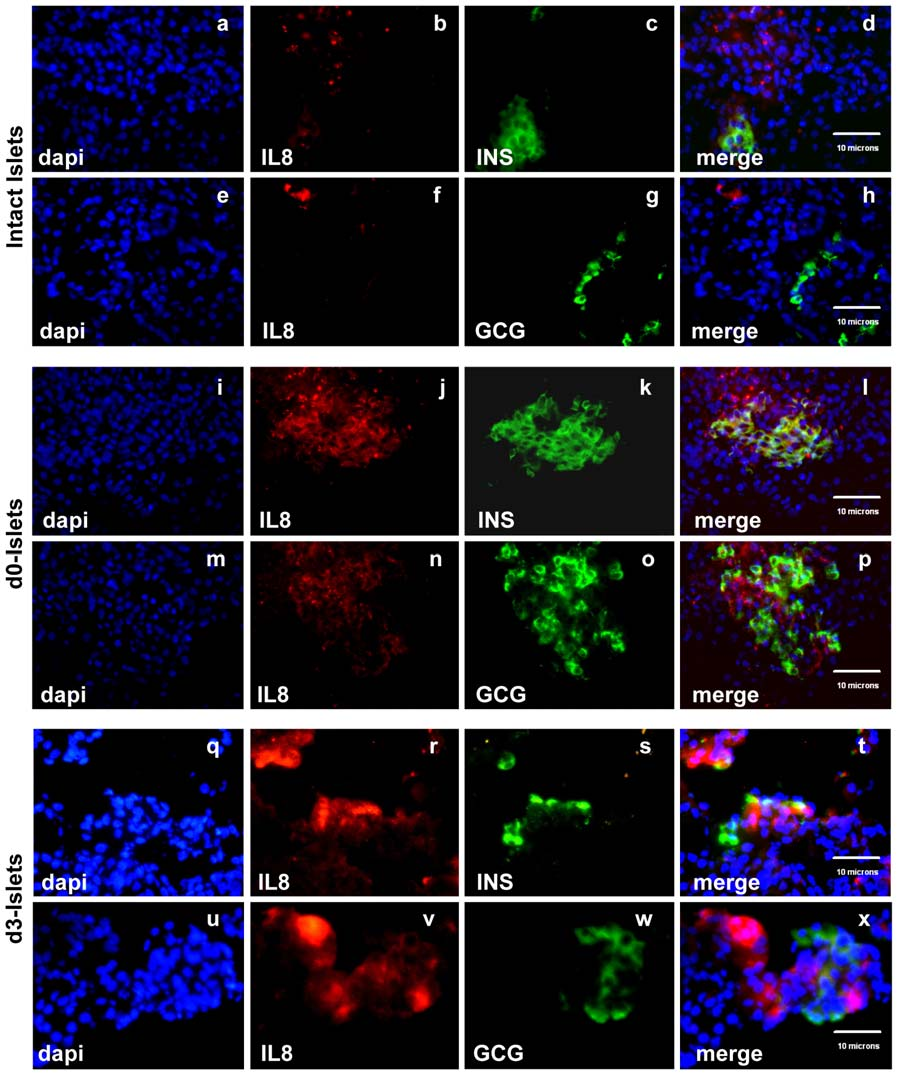
IL-8 expression in isolated islets. Immunofluorescence staining was performed for IL8 in frozen sections from pancreas (a–h), d0-islets (i–p) and d3-islets (q–x) and costaining was performed with insulin (c, k, s) or glucagon (g, o, w). IL-8 expression increases after islet isolation and culture. Most of IL-8 positive cells also costain with insulin but not glucagon. Scale bar (10 micron) on merged image represents scale for all images on its left side.

**Figure 5:**
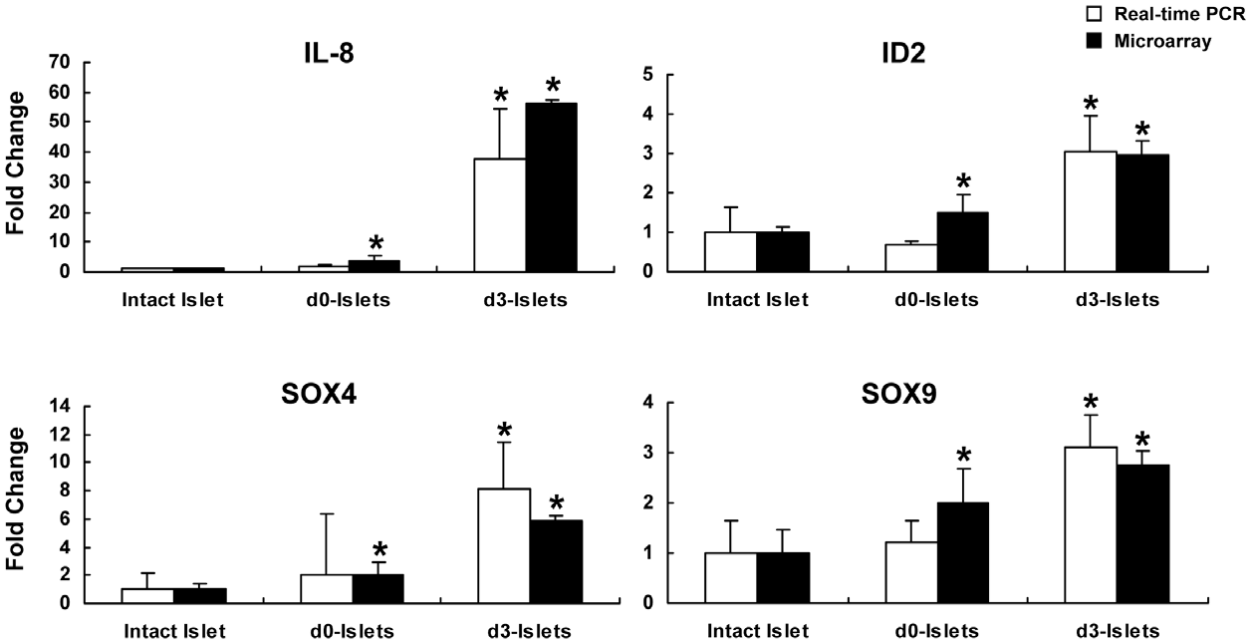
Validation of microarray data by quantitative real-time PCR. Quantitative real-time PCR was performed for IL-8, ID2, SOX4 and SOX9. Fold-changes represent expression level of genes in isolated islets relative to intact islets. Mean fold changes derived from quantitative PCR (white bars) were plotted along with the corresponding mean fold changes from microarray analysis (solid bars). Error bars represent standard deviation. * represents t-test p-values<0.05. For microarray standard deviation and t-test was performed on the expression values obtained after normalization with Lumi. The mean fold changes determined by quantitative PCR were in concordance with the mean fold changes from microarray data.

The secreted cytokine IL-8 was among the most highly induced genes after isolation (3.4-fold induction) and was the most highly upregulated gene in d3 cultured islets (56-fold induction). To confirm IL-8 expression at the protein level, sections of pancreas and isolated islets were stained for IL-8. We found that d3-islets showed intense IL-8 staining as compared with d0-islets (Figure 4). Since our microarray analysis was performed using beta-cell enriched RNA, we examined whether IL-8 protein was in fact being expressed by beta-cells. We found that most insulin-positive cells co-stained with IL-8 in freshly isolated islets. A few IL-8-positive cells did not co-localize with insulin or glucagon in d3-islets (Figure 4), indicating that other cell types within the isolated islet preparations also express IL-8, though beta-cells remained the main source of expression. The upregulation of IL-8 was also confirmed by quantitative PCR (Figure 5).

### NFKB is the central mediator in the inflammatory response

Analysis of our microarray data using DAVID uncovered the NFKB signaling cascade as one of the upregulated pathways. The expression data sets for d0-islets and d3-islets were also analyzed by IPA to determine the activated pathways and networks. Most of the canonical pathways activated in beta-cells from d0-islets or d3-islets were involved in inflammatory and stress signaling events. In addition, the top IPA networks in both d0 and d3 datasets also demonstrated a prominent NFKB response (Figure S3), indicating the importance of this pathway in the induction of post-isolation inflammation. We therefore searched for NFKB target genes among our top 15 upregulated genes from both d0-islets and d3-islets as well as differentially expressed cytokines/chemokines from d3-islets. Six of the top 15 upregulated genes are known to be NFKB target genes (Table 4A). In addition, 22/39 differentially expressed cytokines also have NFKB binding sites within their promoter region (Table 4B). These findings suggest that NFKB is mediating the transcription of several genes in human islets in response to isolation and culture.

**Table 4:** NFKB target genes from top 15 upregulated genes and differentially expressed cytokines and chemokines.

A	B
CFBCXCL6IL-8LTBSAT1VCAM1	BMP2C3CCL2CCL20CCL28CXCL1CXCL2CXCL5CXCL6IL10IL1AIL1BIL1RNIL6IL-8INHBAPLAUS100A6SPP1TGFBITNFSF10TNFSF15

(A) Analysis of the top fifteen upregulated genes from both groups (d0 and d3) revealed that 6/15 genes have NFKB binding sites and, (B) among 39 differentially expressed cytokines/chemokines from d3-islets 22 genes have NFKB binding sites.

### Pancreatic transcription factors are differentially expressed in beta-cells following isolation and *in-vitro* culture

Since beta-cells have the potential to dedifferentiate in culture [10], we examined if expression of transcription factors playing a role in beta-cell specification is affected by the islet isolation process. Expression of a number of transcription factors important for determination for beta- and alpha-cell lineage specification such as NEUROD1, MAFA, MAFB, ARX, NKX2.2 and PBX1 was decreased in isolated islets (Table 5). Furthermore, some pancreatic-progenitor cell specific transcription factors like SOX4, SOX9, and ID2 were upregulated in both d0 and d3-islets. Their expression was also confirmed by quantitative PCR (Figure 5). The mesenchymal cell marker vimentin was also induced by 2.5-fold in d3-islets whereas duct cell markers, CK7 and CK19, were induced by 1.4/1.77 and 3.08/5.4-fold in d0/d3 islets respectively. Taken together, these findings strongly suggest a progressive dedifferentiation of beta-cells during the period of observation.

**Table 5:** Differentially expressed pancreas-specific transcription factors.

Gene	KO phenotype^a^	p-value	Fold change d0-Islets	Fold change d3-Islets	Ref.
ASCL2	Placental failure	7.7×10^−3^	0.90	0.20	[51]
ARX	Loss of alpha cells	1.2×10^−4^	0.52	0.31	[52]
PBX1	Pancreatic hypoplasia defective endocrine & exocrine cell differentiation, die before birth	2.0×10^−3^	0.54	0.35	[53]
MYT1	abnormal islet cells, animal die post-natal	6.4×10^−3^	0.70	0.45	[54]
AHR	Glucose intolerance and altered insulin regulation and response	1.2×10^−3^	0.59	0.46	[55]
HHATL^b^	Negative regulator of palmitoylation of sonic hedgehog	2.1×10^−2^	0.97	0.46	[56]
SOX5^b^	Negative regulator of GSIS	4.2×10^−2^	1.02	0.48	[57]
DACH1	Reduced number of islet cell type	5.4×10^−3^	0.88	0.50	[58]
MAFB	Reduced number of insulin, glucagon expressing cells	2.1×10^−2^	1.08	0.51	[59]
NEUROD1	Severe diabetes and reduction in number of endocrine cells	1.3×10^−2^	0.82	0.58	[60]
FEV^b^	expressed in earliest progenitor cells, known for 5-HT neurons differentiation	5.5×10^−2^	0.61	0.64	[61]
MAFA	Glucose intolerance and impaired GSIS	1.4×10^−3^	0.84	0.69	[42]
ONECUT2	double knock out with HNF6 showed redundant role in differentiation of endocrine precursor	3.8×10^−3^	1.11	1.32	[62]
NOTCH2	Reduced proliferation of pancreatic progenitors and fewer islets	1.4×10^−4^	1.06	1.39	[63]
KIT	lack of functional c-Kit receptors affected beta-cell mass and disrupted beta-cell maturation and function	1.1×10^−2^	1.11	1.54	[64]
FGF2^b^	Stimulates clustering of precursor cells into islet-like aggregates	8.3×10^−3^	1.03	1.61	[47]
SNAI2^b^	Mediator of EMT & expressed in pancreatic progenitor and adult beta and delta cells	3.4×10^−2^	0.93	2.57	[65]
SOX9	Pancreatic hypoplasia, depletion of the progenitor cell pool	7.3×10^−5^	2.01	2.75	[66]
ID2^b^	Promotes expansion of pancreatic progenitors	1.9×10^−7^	1.52	2.94	[48]
SOX4	Reduced number of endocrine cells	8.4×10^−7^	2.05	5.83	[44]

Knock out phenotype, ANOVA p-values and fold changes of differentially expressed pancreas-specific transcription factors.

a KO phenotype : knock out phenotype.

b knock out study is not available, function has been established by other methods.

## Discussion

Maintaining functional beta-cell mass after islet isolation is a critical step for successful and efficient islet transplantation. In spite of several technical advances, the human islet isolation procedure still results in moderate islet recovery and islet recovery following any period of culture is often further reduced [15]. Moreover, the cellular response of pancreatic endocrine cells to isolation and culture remains unclear. The present study aimed to characterize the genome-wide transcriptional response of beta-cells to isolation and culture by comparing gene expression of beta-cells captured from freshly isolated islets (d0) and islets cultured for 3 days (d3) to those in the intact pancreas. This work differs from previous efforts [12] in that LCM was used to collect RNA at all steps, which assures that sample collection and amplification was consistent between all groups. In addition, we have used two time points following isolation (d0 and d3), to allow us to observe the response of human islet cells to isolation and culture. Analysis of the global expression profiles of the samples by PCA and hierarchical clustering revealed a strong correlation of expression data with sample type, indicating that both isolation and culture of human islets induce specific, reproducible changes in global gene expression patterns of human beta-cells.

Functional clustering of differentially expressed genes revealed upregulation of genes associated with inflammation, apoptosis, cell-growth and angiogenesis after islet isolation and culture, suggesting islet exposure to stressful stimuli during the isolation procedure. Concordantly, the top 10 most highly induced genes in both fresh and cultured islets were associated with inflammation and apoptosis. Culturing islets for 3 days induced expression of more mediators of inflammation and apoptosis as well as mediators of oxidative stress, carbohydrate metabolism and NFKB pathways. It is not clear if the expression of these genes in d3-islets was triggered due to stimuli during isolation or culture. It had been suggested that islets are exposed to variety of stresses from culture medium [5], and these may include pro-inflammatory mediators released by islets [16] and enzymes secreted from contaminating exocrine tissue [17]. All these stresses together may contribute to augment inflammatory or apoptotic pathways within cultured islets. Moreover, the nutrient and oxygen supply of cultured islets is solely dependent on diffusion from the medium. Therefore the central beta-cell enriched core might suffer from hypoxic conditions [18] resulting in upregulation of genes associated with oxidative stress. In response to hypoxia, cells enhance oxygen delivery by activating genes associated with angiogenesis and glucose metabolism but persistent hypoxia finally activates cell death pathways [19]. Therefore, the observed upregulation of mediators of cell growth, angiogenesis and glucose metabolism could be part of the hypoxia response. Cultured islets also showed downregulation of genes associated with response to insulin and glucose stimuli. This is consistent with the observation that culturing islets under normal conditions compromises beta-cell function [20].

Islet cells produce various cytokines in response to metabolic or immunological stresses which promote repair and adaptation to these stimuli. Resident or infiltrating macrophages, ductal cells, beta-cells and endothelial cells have all been shown to be capable of producing inflammatory cytokines [21], [22], the increased levels of which have been linked to graft function [23]. In the present work, several cytokines and cytokine receptors showed increased expression in the beta-cell enriched samples from fresh or cultured islets. Furthermore, IL-10, a negative regulator of cytokine expression was downregulated by 0.55 and 0.38-fold in d0 and d3-islets respectively. IL-8, a potent inflammatory mediator and an angiogenic factor, was the most highly induced gene. Interestingly our immunohistochemical studies confirmed that the majority of IL-8 was produced by beta-cells in isolated human islets. Other studies have observed IL-8 production from alpha-cells of type II diabetic patients and from non-endocrine fractions of islet preparations [21], [24]. While we did not observe colocalization of IL-8 and glucagon, we did observe IL-8 staining in non-beta-cells, suggesting additional sources of IL-8 expression. Earlier studies have shown that islet preparations contain resident macrophages, ductal cells and vascular endothelial cells [25] which could be other sources of IL-8 expression in our islet preparations. While the functional consequences of IL-8 production by beta-cells are not known, IL-8 production has been associated with poor outcomes in other transplantation procedures. IL-8 release during organ procurement has also been observed with kidney, lung and liver and has been associated with poor graft function in human liver and lung transplantation [26], [27], [28]. Application of pharmacological modulators or IL-8 antagonist resulted in improved graft function in animal models of lung and renal transplantation [27], [29]. Moreover, IL-8 has also been suggested as a marker of sustained acute renal transplant failure and early graft function after human lung transplantation [30]. Taken together, these findings suggest that suppression of IL-8 signaling may be beneficial to islet graft survival.

Early islet graft loss has been attributed to the IBMIR, during which massive neutrophil infiltration damages transplanted islets and enhances subsequent cell-mediated immune responses [6]. It has been suggested that IBMIR is promoted by expression of TF and MCP1 on islets, [8] as most of the islet cells express these markers [31]. In the present study, we found that MCP1 was induced in d0-islets (1.74-fold) and d3-islets (3.75-fold) whereas TF was induced in d3-islets (1.9-fold). It is important to note that IL-8 is also a potent neutrophil and macrophage chemoattractant and activator. Other neutrophil activators like CXCL1, CXCL2, CXCL6, IL-1 and IL-6 were also upregulated in both d0 and d3-islets. Therefore, our findings highlight additional mediators of neutrophil migration, which may play a role in IBMIR.

Pathway analysis of array data revealed activation of inflammatory and stress signaling canonical pathways in beta-cell enriched samples of islets and suggests NFKB as a central mediator of the inflammatory response in both fresh and cultured islets. Most of the 15 highly induced genes and differentially expressed cytokines from d0 and d3-islets have NFKB binding sites in their promoter regions and have been previously established as NFKB target genes. NFKB activity is induced by various stimuli like cytokines, viral proteins and stress inducers and is known to regulate expression of several target genes critical for the stress response, cell growth, survival and apoptosis [32]. Indeed, our finding that isolated islets express high levels of cytokines and have reduced expression of IL10, a known suppressor of NFKB activation [33], suggests that NFKB is activated in isolated islets in response to an altered cytokine milieu. Indeed, it had been observed that NFKB DNA binding activity, indicative of pathway activation, increases in islets after isolation and even 2 days later in cultured islets [34] and evidence suggests that inhibition of NFKB activity improves islet graft function [35], [36]. Our findings have uncovered several NFKB target genes which may mediate the negative effect of this pathway on islet survival.

Cell based therapies for diabetes are severely limited by the inability to properly maintain beta-cells in culture, even for periods of less than a week [10]. However, little is known about the processes mediating beta-cell dedifferentiation in culture. Activation of the Notch pathway, which is not normally present in adult human beta-cells, appears to suppress apoptosis as well as promote beta-cell dedifferentiation in culture [37], [38]. We observed a decrease in the expression of beta-cell specific transcription factors upon isolation and culturing of human islets, including key regulators like NEUROD1, MAFA, and MAFB. NEUROD1 is required for maintaining maturity and proper functioning in adult beta-cells and beta-cells lacking NEUROD1 resemble immature beta-cells [39]. MAFB is required for beta-cell maturation [40], whereas MAFA is essential for maintenance and function of adult beta-cells [41]. Knock down of MAFA or MAFB results in reduced numbers of insulin producing cells and poor islet function [40], [42]. Transcription factors found in endocrine progenitor cells, like SOX4, SOX9, and ID2 were induced during isolation process. The Sox family of transcription factors play a critical role in the maintenance of the pancreatic progenitor cell pool and the development and function of islet cells [43]. SOX4 and SOX9 are required for the maintenance of undifferentiated progenitor cells and endocrine cell pool [44], [45]. Interestingly, there is evidence that SOX9 expression is induced by NFKB in a chondrogenic cell line [46], suggesting that such a mechanism could be occurring in isolated islets. FGF2, one of the over expressed factor, is also expressed by pancreatic precursor cells and promotes clustering to form islet-like aggregates [47]. ID2 is a negative regulator of cell differentiation and may have an important role in expansion of pancreatic progenitor cell [48]. Moreover, expression of Vimentin, a mesenchymal cell marker and the duct-lineage cell markers, CK7 and CK19, also are progressively induced in islets. A recent study on adult human islets revealed the presence of a heterogeneous population of beta-cells with different maturation states [49]. The authors identified a population of cells expressing multiple hormones, as well as other genes associated with pancreas development, suggesting that they are immature beta-cells. It is interesting to note that several genes enriched in these immature cells were also found to be upregulated in cultured islets in our study. These findings suggested that the beta-cell specific transcriptional network is affected during isolation process, and *in-vitro* culture further induces de-differentiation of mature beta-cell towards pancreatic progenitor cell stage.

Certain technical limitations need to be considered in the analysis of LCM-derived data. Although careful attention was paid in selecting only autofluorescent beta-cells, the presence of other endocrine cell types is inevitable given the scattered architecture and close proximity of endocrine cell types within human islets [50]. However, the presence of other endocrine cells in all three sample types was expected to be proportionate and consistent, thus still making our samples comparable.

In conclusion, our study provides new insights into the implications of islet isolation and culture on beta-cells. In summary, beta-cells in isolated human islets display signs of inflammation, metabolic changes, and dedifferentiation that could combine to reduce functional beta-cell mass. These pathways may also adversely impact cell engraftment and function following transplantation. Application of cytokine antagonists, especially to IL-8, or of NFKB modulators during isolation or before transplantation may have beneficial effects on beta-cell survival and function and could ultimately improve transplantation outcomes.

## Supporting Information

Figure S1 **Colocalization of beta-cell autoflourescence signal with insulin.** Intact pancreatic islets (a, d) and isolated islets (g. j) were visualized for their autofluorescence signal and immunostained with insulin (b, h) or glucagon (e, k) and images were merged (c, f, i, l). Autofluorescence signal colocalized with insulin.
(TIF)

Figure S2 **LCM images and RNA quality.** Beta-cells were identified by their intrinsic autofluorescence and captured by LCM. Beta-cells within the intact pancreas (a–f) and d0-islets (g–l) and d3 islets (m–r) were observed with either bright field or fluorescence. Islet before LCM (a, d, g, j, m, p); islet after LCM (b, e, h, k, n, q); captured cells on the cap (c, f, i, l, o, r). Electropherogram for RNA extracted from beta-cells enriched tissue by LCM from islets from the intact islets (s) d0- islets (t) and d3-islets (u). RIN: RNA integrity number
(TIF)

Figure S3 **Ingenuity Pathways Assist analysis.** IPA identified NFKB dependent processes as top networks in isolated islets; (A) top network for d0-islets and (B) top network for d3-islets. It suggests that NFKB activity contributes to the inflammatory process observed following islet isolation and in vitro culture. Colored boxes represent genes differentially expressed in our dataset with red representing upregulation and green representing downregulation (different shades of the color signify the degree of expression). Direct relationships are shown as solid arrows and indirect relationships are shown as dashed arrows. Arrows highlighted in blue denote NFKB dependent relationships.
(TIF)

Table S1 Differentially expressed cytokines/chemokines and their receptors in d0-islets and d3-islets with their ANOVA p-values and fold-changes relative to intact islets.
(DOC)

Table S2 List of genes and primers used for quantitative Real-time PCR.
(DOC)
