# Supplementary figures and images for: Analysis of Beta-Cell Gene Expression Reveals Inflammatory Signaling and Evidence of Dedifferentiation following Human Islet Isolation and Culture
Source: PLoS One. 2012 Jan 27;7(1):e30415. doi: 10.1371/journal.pone.0030415 (PMC3267725; doi:10.1371/journal.pone.0030415)

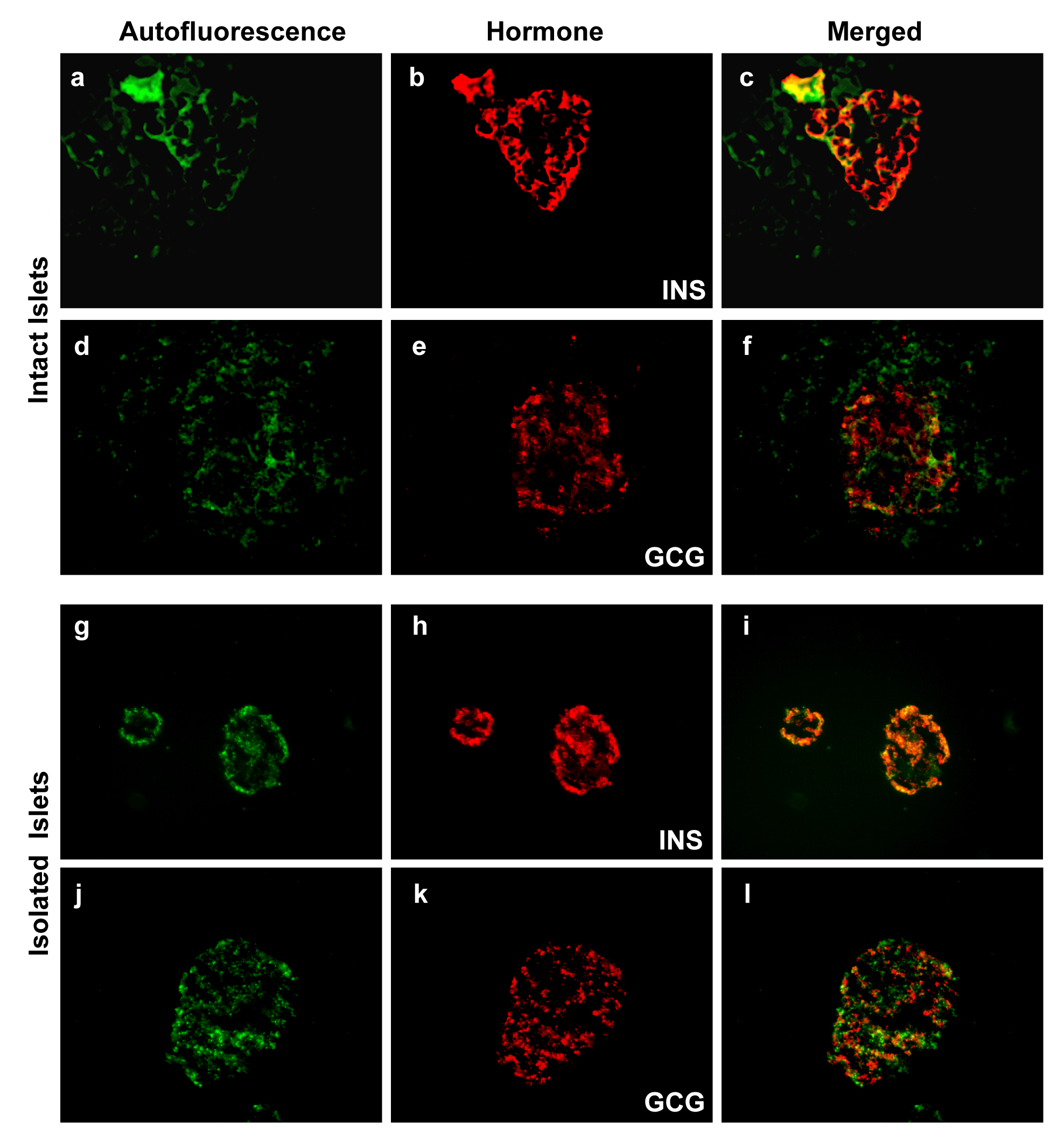

Supplement: Figure S1 — Colocalization of beta-cell autoflourescence signal with insulin. Intact pancreatic islets (a, d) and isolated islets (g. j) were visualized for their autofluorescence signal and immunostained with insulin (b, h) or glucagon (e, k) and images were merged (c, f, i, l). Autofluorescence signal colocalized with insulin. (TIF) [file pone.0030415.s001.tif]

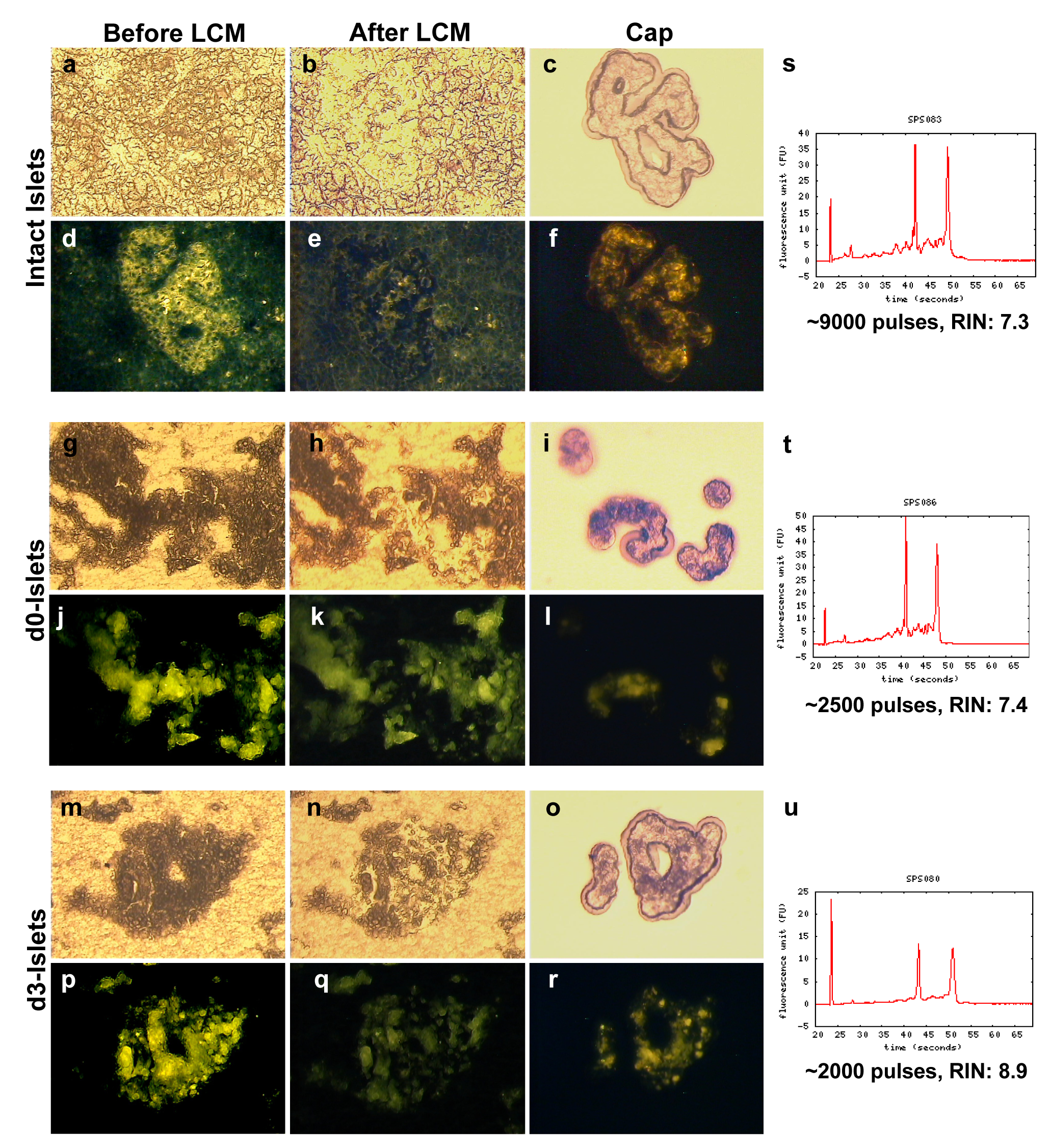

Supplement: Figure S2 — LCM images and RNA quality. Beta-cells were identified by their intrinsic autofluorescence and captured by LCM. Beta-cells within the intact pancreas (a–f) and d0-islets (g–l) and d3 islets (m–r) were observed with either bright field or fluorescence. Islet before LCM (a, d, g, j, m, p); islet after LCM (b, e, h, k, n, q); captured cells on the cap (c, f, i, l, o, r). Electropherogram for RNA extracted from beta-cells enriched tissue by LCM from islets from the intact islets (s) d0- islets (t) and d3-islets (u). RIN: RNA integrity number (TIF) [file pone.0030415.s002.tif]

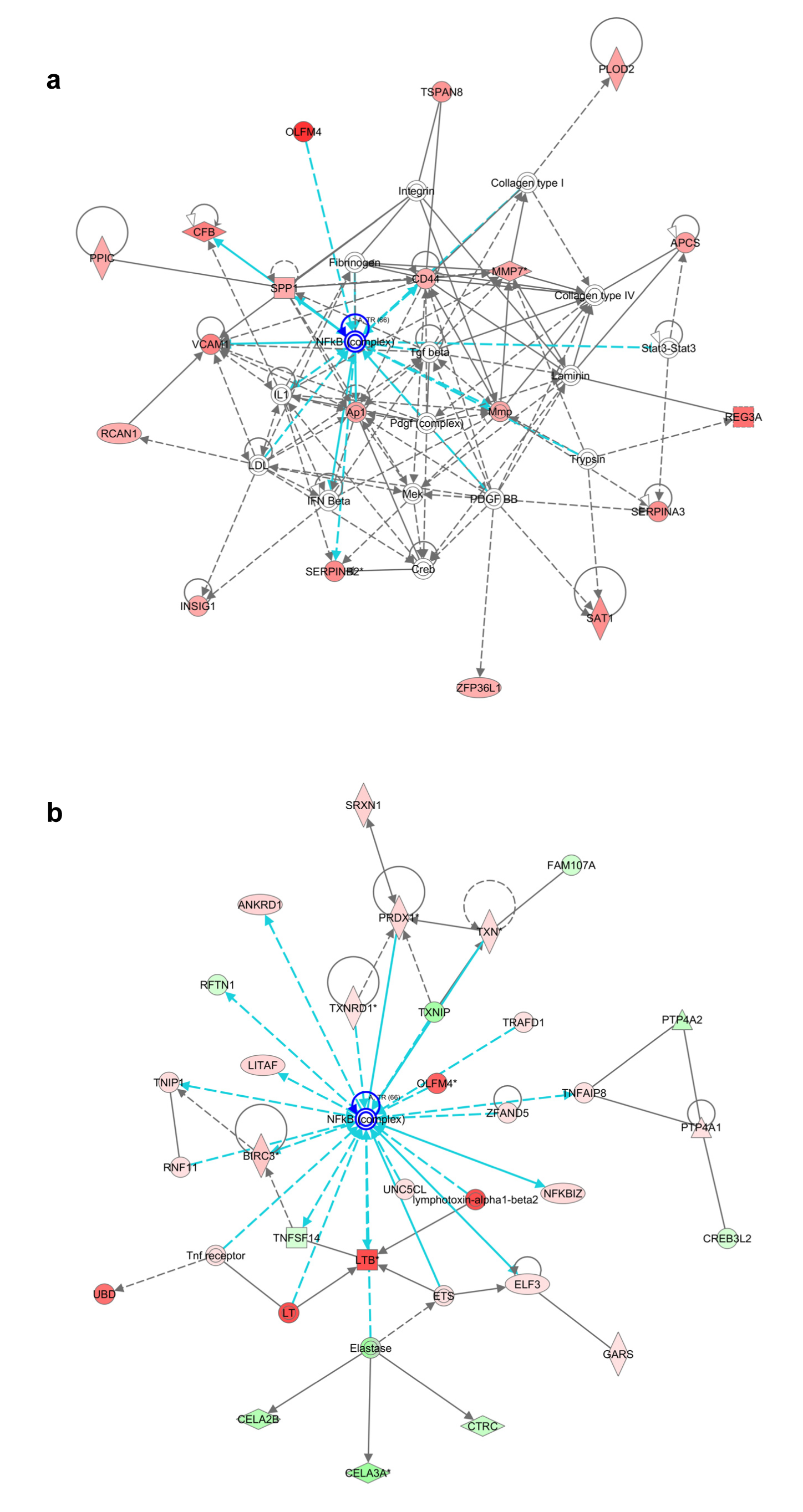

Supplement: Figure S3 — Ingenuity Pathways Assist analysis. IPA identified NFKB dependent processes as top networks in isolated islets; (A) top network for d0-islets and (B) top network for d3-islets. It suggests that NFKB activity contributes to the inflammatory process observed following islet isolation and in vitro culture. Colored boxes represent genes differentially expressed in our dataset with red representing upregulation and green representing downregulation (different shades of the color signify the degree of expression). Direct relationships are shown as solid arrows and indirect relationships are shown as dashed arrows. Arrows highlighted in blue denote NFKB dependent relationships. (TIF) [file pone.0030415.s003.tif]
