# Supplementary material for: Analysis of Beta-Cell Gene Expression Reveals Inflammatory Signaling and Evidence of Dedifferentiation following Human Islet Isolation and Culture
Source: PLoS One. 2012 Jan 27;7(1):e30415. doi: 10.1371/journal.pone.0030415 (PMC3267725; doi:10.1371/journal.pone.0030415)
Supplement: Table S1 — Differentially expressed cytokines/chemokines and their receptors in d0-islets and d3-islets with their ANOVA p-values and fold-changes relative to intact islets. (DOC) [file pone.0030415.s004.doc]

**Table S1: Differentially expressed cytokines/chemokines and their receptors in d0-islets and d3-islets with their ANOVA p-values and fold-changes relative to intact islets**

| **Gene** | **p-Value** | **Fold change (d0-Islet)** | **Fold change (d3-Islet)** |
| --- | --- | --- | --- |
| **Cytokines/Chemokines** | | | |
| IL-8 | 6.4x10^-7^ | 3.4 | 56 |
| CXCL6 | 5.8x10^-7^ | 1.6 | 30.2 |
| LTB | 4.5x10^-5^ | 1.7 | 14.3 |
| SPP1 | 3.8x10^-4^ | 1.9 | 8.2 |
| ADM | 2.2x10^-4^ | 1.9 | 5.2 |
| IL33 | 5.2x10^-3^ | 1.0 | 4.6 |
| CXCL2 | 2.3x10^-4^ | 1.5 | 4.5 |
| CXCL1 | 2.9x10^-4^ | 1.2 | 4.3 |
| NAMPT | 5.0x10^-5^ | 1.4 | 4.3 |
| IL32 | 2.2x10^-2^ | 1.3 | 4.0 |
| S100A6 | 2.8x10^-5^ | 1.6 | 3.9 |
| CCL2 | 1.0x10^-2^ | 1.7 | 3.8 |
| IL1A | 1.1x10^-2^ | 1.0 | 3.7 |
| PLAU | 1.1x10^-4^ | 1.1 | 3.3 |
| TGFBI | 1.3x10^-3^ | 1.1 | 3.2 |
| GREM1 | 2.1x10^-4^ | 1.3 | 3.1 |
| STC1 | 2.0x10^-4^ | 1.2 | 3.0 |
| CXCL16 | 8.9x10^-3^ | 1.6 | 2.6 |
| IL24 | 3.0x10^-3^ | 1.3 | 2.5 |
| BMP2 | 7.1x10^-4^ | 1.4 | 2.4 |
| TNC | 2.6x10^-4^ | 1.1 | 2.3 |
| C3 | 2.0x10^-3^ | 1.2 | 2.1 |
| CKLF | 1.1x10^-2^ | 1.7 | 2.1 |
| CXCL5 | 5.1x10^-3^ | 1.0 | 2.1 |
| TNFSF10 | 9.9x10^-3^ | 1.1 | 2.1 |
| CCL20 | 3.1x10^-2^ | 1.0 | 2.0 |
| CCL28 | 9.5x10^-4^ | 1.2 | 2.0 |
| GMFB | 1.1x10^-2^ | 1.8 | 1.9 |
| IL1B | 3.8x10^-2^ | 1.0 | 1.8 |
| IL6ST | 3.5x10^-2^ | 1.1 | 1.8 |
| TGFA | 6.6x10^-3^ | 1.3 | 1.8 |
| IL1RN | 2.1x10^-3^ | 1.1 | 1.7 |
| INHBA | 1.2x10^-4^ | 1.0 | 1.7 |
| IL6 | 3.5x10^-2^ | 1.0 | 1.5 |
| TNFSF15 | 8.8x10^-5^ | 0.8 | 0.6 |
| IL18 | 4.3x10^-4^ | 0.7 | 0.5 |
| TNFSF14 | 2.3x10^-3^ | 0.6 | 0.5 |
| BMP8B | 2.5x10^-4^ | 0.6 | 0.4 |
| IL10 | 2.4x10^-4^ | 0.6 | 0.4 |
| **Cytokine Receptors** | | | |
| TNFRSF10B | 3.0x10^-4^ | 2.0 | 3.7 |
| EGFR | 2.4x10^-3^ | 1.8 | 3.5 |
| TNFRSF11B | 2.6x10^-6^ | 1.4 | 2.8 |
| ACVR1 | 5.1x10^-3^ | 1.4 | 2.5 |
| EDNRA | 2.5x10^-2^ | 1.2 | 2.5 |
| IFNAR2 | 4.4x10^-4^ | 1.3 | 2.4 |
| IL13RA1 | 1.0x10^-3^ | 1.3 | 2.3 |
| IL10RB | 3.1x 10^-3^ | 1.2 | 2.2 |
| ANGPTL2 | 1.2x10^-2^ | 1.3 | 2.0 |
| TNFRSF1A | 7.5x10^-3^ | 1.2 | 2.0 |
| IFNAR1 | 3.5x10^-2^ | 1.2 | 1.9 |
| IL13RA2 | 3.0x10^-2^ | 1.1 | 1.9 |
| IFNGR1 | 3.7x10^-4^ | 1.5 | 1.8 |
| IFNGR2 | 4.1x10^-2^ | 1.2 | 1.8 |
| IL17RB | 4.5x10^-3^ | 1.0 | 1.6 |
| GPR44 | 9.9x10^-3^ | 0.9 | 0.5 |
| IL17RD | 5.8x10^-5^ | 0.8 | 0.5 |
| CSF2RA | 1.1x10^-4^ | 0.6 | 0.4 |
