# Supplementary material for: Analysis of Beta-Cell Gene Expression Reveals Inflammatory Signaling and Evidence of Dedifferentiation following Human Islet Isolation and Culture
Source: PLoS One. 2012 Jan 27;7(1):e30415. doi: 10.1371/journal.pone.0030415 (PMC3267725; doi:10.1371/journal.pone.0030415)
Supplement: Table S2 — List of genes and primers used for quantitative Real-time PCR. (DOC) [file pone.0030415.s005.doc]

**Table S2**

**List of genes used for quantitative Real-time PCR**

| **Transcript** | **Gene Bank Accession No.** | **Primer sequence** |
| --- | --- | --- |
| IL-8 | NM_000584 | Forward GAGTGGACCACACTGCGCCA  Reverse TCCACAACCCTCTGCACCCAGT |
| ID2 | NM_002166 | Forward ACGCCGCTGACCACCCTCAA  Reverse CCGCTTATTCAGCCACACAGTGC |
| SOX4 | NM_003107 | Forward GGCCTGTTTCGCTGTCGGGT  Reverse GCCTGCATGCAACAGACTGGC |
| SOX9 | NM_000346 | Forward GCGGACCAGTACCCGCACTT  Reverse TTCACCGACTTCCTCCGCCGC |
| RPS16 | NM_001020 | Forward GGCAATGGTCTCATCAAGGT  Reverse TCTCCTTCTTGGAAGCCTCA |
| RPS18 | NM_022551 | Forward CCAAGAGGGCGGGAGAACTCA  Reverse ACCATTGGCTAGGACCTGGCTG |
